# P-1758. Evaluation of Real-Life Application of Multi-plex PCR Pneumonia Film-array Panel as an Antibiotic Stewardship Tool, a Prospective Investigation

**DOI:** 10.1093/ofid/ofae631.1921

**Published:** 2025-01-29

**Authors:** Hera Maryam, Brian Kim, Niki Arab, Christian Olivo Freites, Daisuke Furukawa, Oscar Gallardo-Huizar, Glen Huang, Arthur Jeng

**Affiliations:** UCLA, Los Angeles, California; Olive View-UCLA Medical Center, Sylmar, California; Olive View- UCLA Medical Center, Los Angeles, California; Ryan Health, New York, New York; Stanford University, Palo Alto, California; UCLA, Los Angeles, California; University of California - Los Angeles, Los Angeles, California; Olive View UCLA Medical Center/UCLA School of Medicine, Sylmar, California

## Abstract

**Background:**

Selecting empiric antibiotics (abx) for community-acquired and hospital-acquired pneumonia (CAP, HAP) on admission can be challenging, given the variety of possible pathogens. Per IDSA guidelines, broad spectrum antibiotic(s) are recommended, based on pneumonia type, risk of prior abx exposure/drug resistance, and disease severity. In this study, by using the BioFire® multi-plex PCR film-array (FA) on sputa, we prospectively investigated the time to pathogen identification in CAP and HAP, time to providing optimal abx, and types of change this strategy allowed.

**Figure:**
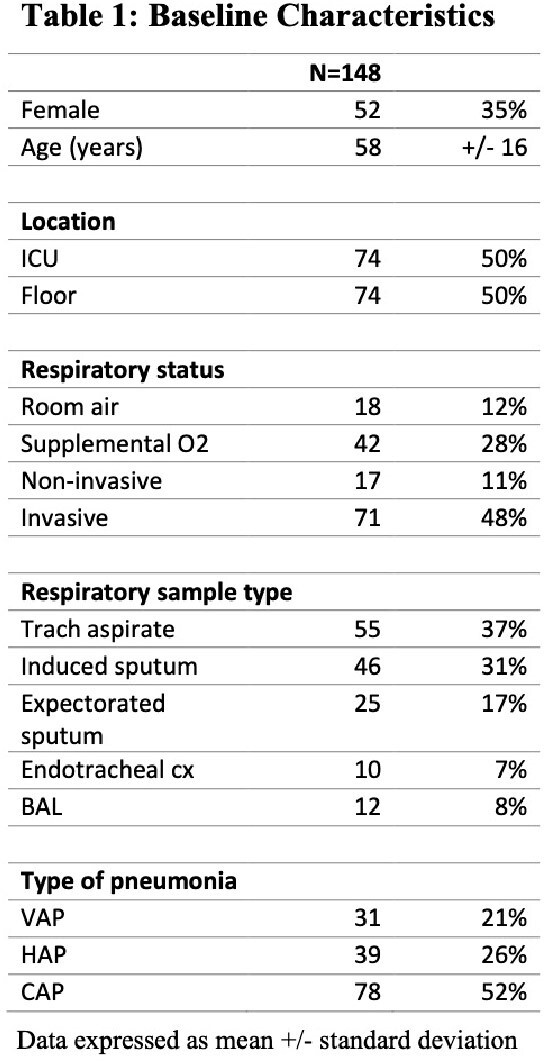

**Methods:**

Single center, prospective, observational study from January 1, 2021 to January 31, 2024. All admitted patients with CAP or HAP, with satisfactory sputa or bronchioalveolar lavage (BAL) cultures, were considered for inclusion by the Antimicrobial Stewardship service (ASP), whereby FA was performed on the sample. Exclusion criteria consisted of poor sample (≥ 10 epithelial cells/lpf), sputa previously ordered within 7 days, concurrent non-pneumonia infections and non-infectious pulmonary conditions. FA results and its respective abx recommendations were communicated to the patient’s clinician by ASP.

**Figure:**
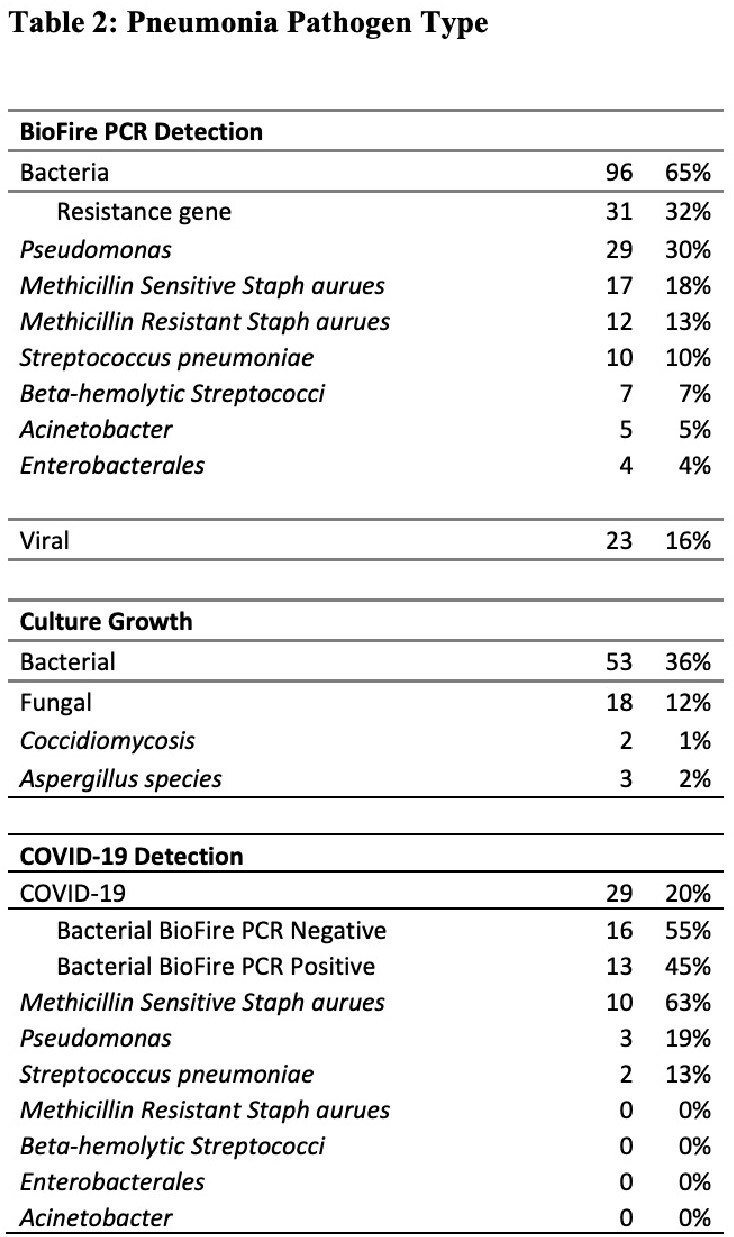

**Results:**

148 samples met study criteria (Table 1). FA detected bacterial pathogens in 65% (n=96) and viruses in 16% (n=23) (Table 2). FA resulted in 2.9 hours (H) [IQR 1.75-2.87] after gram stain, whereas time to culture finalization was 90.5 H [IQR 45-82] (Figure 1). An average of 2 empiric abx days were saved per case [IQR 1-2]. Time to first modification of abx was 6.1 H [IQR 0.15-2.9]. 84% of abx courses were modified due to ASP intervention from FA, of which 86% resulted in de-escalation of empiric abx (Figure 2). 62% (n=92) had no growth on culture, and of those cases, 48% (n=44) had pathogen detected by FA. 50% (n=74) of bacterial culture results were congruent to FA, 19% (n=28) were partially congruent, with more bacteria detected on FA than culture.

**Figure:**
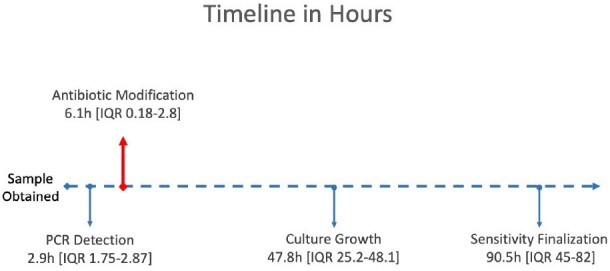

Timeline

**Conclusion:**

FA reduced time to pathogen identification compared to traditional culture in CAP and HAP, allowing ASP to provide rapid interventions to optimize abx treatment and save days of less optimal empiric abx. FA detected more pathogens compared to culture, including when negative, leading to abx changes in majority of cases. FA serves as a valuable tool for ASP.

**Figure:**
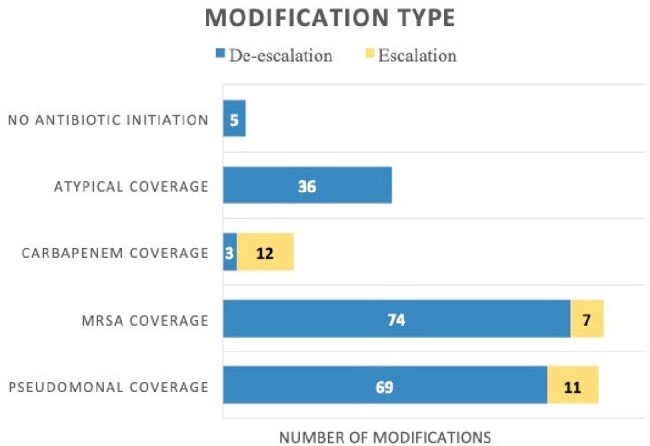

Type of Intervention

**Disclosures:**

**All Authors**: No reported disclosures

